# Rapid CD4^+^ T-cell responses to bacterial flagellin require dendritic cell expression of Syk and CARD9

**DOI:** 10.1002/eji.201444744

**Published:** 2014-12-08

**Authors:** Shaikh M Atif, Seung-Joo Lee, Lin-Xi Li, Satoshi Uematsu, Shizuo Akira, Sara Gorjestani, Xin Lin, Edina Schweighoffer, Victor L J Tybulewicz, Stephen J McSorley

**Affiliations:** 1 Center for Comparative Medicine, Department of Anatomy, Physiology, and Cell Biology, University of California DavisDavis, CA, USA; 2 Laboratory of Host Defense, WPI Immunology Frontier Research Center, Osaka University, Suita, OsakaJapan; 3 Department of Mucosal Immunology, School of Medicine, Chiba UniversityChuou-ku, Chiba, Japan; 4 Department of Molecular and Cellular Oncology, The University of Texas MD Anderson Cancer CenterHouston, TX, USA; 5 Medical Research Council, National Institute for Medical ResearchLondon, UK

**Keywords:** Bacterial infection, CARD9, CD4^+^ T cells, Syk, TLRs

## Abstract

Toll-like receptors (TLRs) can recognize microbial patterns and utilize adaptor molecules, such as-MyD88 or (TRIF TIR-domain-containing adapter-inducing interferon-β), to initiate downstream signaling that ultimately affects the initiation of adaptive immunity. In addition to this inflammatory role, TLR5 expression on dendritic cells can favor antigen presentation of flagellin peptides and thus increase the sensitivity of flagellin-specific T-cell responses in vitro and in vivo. Here, we examined the role of alternative signaling pathways that might regulate flagellin antigen presentation in addition to MyD88. These studies suggest a requirement for spleen tyrosine kinase, a noncanonical TLR-signaling adaptor molecule, and its downstream molecule CARD9 in regulating the sensitivity of flagellin-specific CD4^+^ T-cell responses in vitro and in vivo. Thus, a previously unappreciated signaling pathway plays an important role in regulating the dominance of flagellin-specific T-cell responses.

## Introduction

Innate immune receptors are part of the first line of host defense and protect against foreign pathogens [1,2]. The surface of innate immune cells is decorated with multiple receptors, including pattern-recognition receptors (PRRs) and scavenging receptors, that together can recognize an invading pathogen, generate a local inflammatory response. PRRs are able to sense the presence of conserved microbial molecules, including proteins, lipopolysaccharides, lipoproteins, and nucleic acids [1,2]. Importantly, PRR-mediated ligand recognition can modulate the activation status of dendritic cells (DCs) and the subsequent ability of this population to initiate an adaptive immune response [3,4]. Although there are 13 different Toll-like receptors (TLRs) described in humans [5], only TLR5 and TLR11 predominantly recognize protein ligands [6,7]. Since host T cells also directly recognize protein fragments, this creates a unique situation where a TLR and the responding T-cell population can directly recognize the same microbial ligand. It is currently unclear how this overlapping innate immune response to the same protein ligand might affect the quantity and quality of the adaptive immune response to TLR5 or TLR11 ligands.

TLR ligation initiates a cascade of signaling events that utilizes several different intracellular adapter proteins. With the exception of TLR3, which requires TIR-domain-containing adapter-inducing interferon-β, all TLRs can initiate downstream signals through myeloid differentiation factor 88 (MyD88), while TLR4 can independently engage both MyD88 and TIR-domain-containing adapter-inducing interferon-β [8]. TLR5 is expressed by a variety of cell types and is known to regulate innate and adaptive immune responses to bacterial flagellins [9–12]. Binding of flagellin to TLR5 initiates receptor dimerization, allowing the TIR domains of TLR5 to interact with the TIR domain of MyD88. MyD88 subsequently transmits this signal by initiating a cascade of signaling events that recruits interleukin-1 receptor-associated kinase (IRAK)1/4, TAK, TBK, MEK, JNK to ultimately induce the transcription of genes through NF-kB or AP-1 [8].

C-type lectin receptors (CLRs) are another class of PRRs that appear to primarily recognize fungal components and can also regulate host inflammatory responses and influence infection-associated pathology [13]. Signaling downstream of CLR ligation is most clearly understood for Dectin-1, a receptor that recognizes fungal β-glucans [14]. Dectin-1 ligation induces MyD88-independent phosphorylation of an immune receptor tyrosine-based activation-like motif (ITAM-like) and recruits and activates spleen tyrosine kinase (Syk) that ultimately causes downstream activation of NF-kB [15–17]. Besides TLRs and CLRs, Nod-like receptors (NLRs) are an additional class of PRR that function as intracellular sensors of pathogens that can regulate pathways independently of MyD88 and induce NF-kB and the generation of proinflammatory molecules [18]. Recently, Syk has also been shown to regulate inflammasome-mediated formation of adaptor protein ASC (apoptosis associated speck-like protein containing a CARD), an important step that controls responses via NLRs. Therefore, signals through Syk can regulate the function of CLRs and additionally affect NLRP3 (NACHT, LRR and PYD domain containing protein 3) inflammasome-mediated IL-18 secretion and caspase-1 activity in macrophages [19]. However, Syk is largely dispensable for NACHT, LRR and PYD domain containing protein 3 inflammasome activation in DCs, suggesting that Syk can play different roles in different immune cell types [20].

Flagellin is the primary protein component of bacterial flagella, a whip-like appendage that is found on the surface of both gram-positive and gram-negative bacteria and assists bacterial locomotion and adherence to host epithelium [21]. Flagellin is directly recognized by TLR5 to generate an inflammatory response and can also elicit a robust adaptive immune response even when nanograms quantities are injected into an animal [6,22,23]. Although flagellin can also be recognized by the intracellular sensors Naip5 and NLRC4 [24], the ability of flagellin to initiate a robust adaptive immune response at very low concentrations has been shown to be highly dependent upon the expression of TLR5 [12].

Previous studies in our laboratory have demonstrated that TLR5 is required but that MyD88 is dispensable for the induction of CD4^+^ T-cell activation responses to low concentrations of flagellin [12], however, it is currently unclear how such responses via TLR5 occur in the absence of MyD88. Here, we demonstrate Syk and CARD9 signaling pathways can influence TLR5-mediated antigen presentation and that Syk and CARD9 expression are required for optimal activation of flagellin-specific CD4^+^ T cells. These data suggest the presence of signaling pathways that are downstream of TLR5 or another coreceptor that can participate in antigen presentation of flagellin epitopes.

## Results

### TLR5 is required for robust initial activation of flagellin-specific T cells

**Figure 1:**
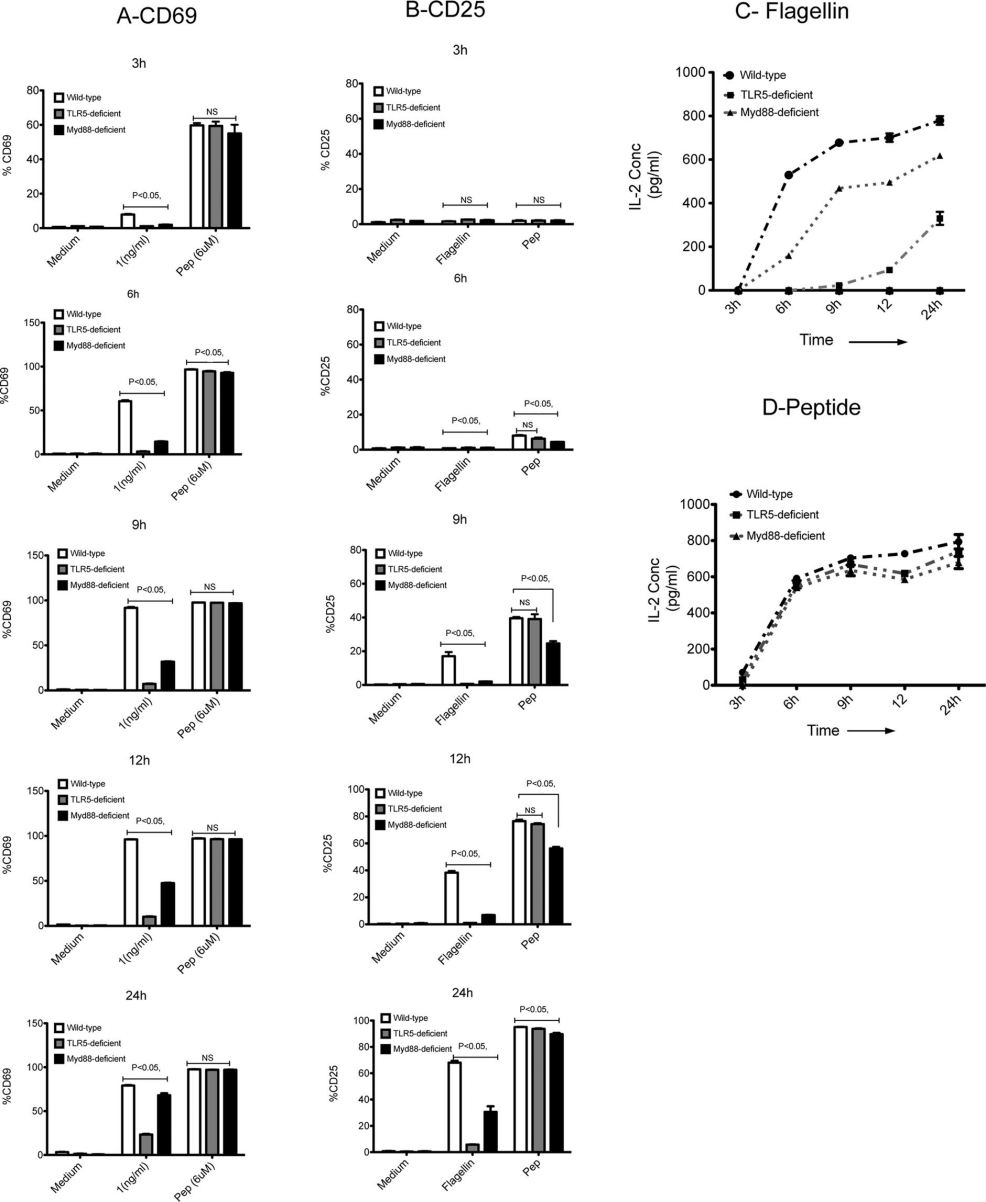
TLR5 is essential for activation of flagellin-specific T cells at low dose of flagellin. Time kinetics was performed to evaluate the activation of flagellin-specific SM1 T cells (CD4^+^CD90.1^+^) in vitro, using CD69 and CD25 as markers of activation. CD11c^+^ DCs were purified from spleens of WT (white bars), TLR5-deficient (gray bars), and MyD88-deficient DCs (black bars), using DC enrichment kits and were cultured in 1:1 ratio with flagellin-specific SM1 T cells in the presence of flagellin (1 ng/mL), peptide (6 μM), and medium alone for 3, 6, 9, 12, and 24 h. (A and B) The activation of flagellin-specific T cells as determined by (A) CD69 expression and (B) CD25 expression at given time points, was measured by flow cytometry. Data are shown as mean + SEM of three samples per group, and are from one experiment, representative of two independent experiments. (C and D) Concentration of IL-2 in DC culture supernatants at 3, 6, 9, 12, and 24 h, as measured by ELISA. CD11c^+^ DCs purified from spleens of WT (filled circles), TLR5-deficient (filled squares), and MyD88-deficient DCs (filled triangles) using DC enrichment kits and were cultured in 1:1 ratio with flagellin-specific SM1 T cells in the presence of (C) flagellin (1 ng/mL) or (D) peptide (6 μM) for 3, 6, 9, 12, and 24 h. Data are shown as mean ± SEM of three samples per group carried out in tissue culture replicates, and are from one single experiment representative of two independent experiments. NS: nonsignificant by unpaired *t*-test.

Previous reports have shown that TLR5-deficient DCs have impaired ability to activate flagellin-specific SM1 T cells when flagellin protein is used as an antigen [12]. Importantly, TLR5-deficient and wild-type (WT) DCs displayed equivalent ability to activate SM1 T cells in response to a wide range of flagellin peptide, or OT-II T cells in response to ovalbumin (OVA) [12]. To examine this phenomenon in more detail, we monitored the ability of WT, TLR5-, or MyD88-deficient splenic DCs to activate flagellin-specific T cells at different time points over a 24-h period. WT, TLR5-, and MyD88-deficient DCs were each able to activate SM1 T cells efficiently when processed flagellin peptide was added to in vitro cultures, causing increased surface expression of CD69 and CD25 and secretion of IL-2 (Fig.1A, B, and D). Thus, the DC populations from TLR5- and MyD88-deficient mice have similar stimulatory ability for SM1 T cells when compared directly to WT DCs. However, when these same DCs were exposed to whole flagellin that requires processing before antigen presentation can occur, a striking requirement for DC expression of TLR5 was observed. Thus, incubation of SM1 T cells with WT DCs and flagellin stimulated increased surface expression of CD69 and CD25 on SM1 T cells within 3 and 9 h, respectively (Fig.1A and B). In contrast SM1 T cells incubated with TLR5-deficient DCs did not increase expression of CD69 or CD25 at all in the first 12 h of culture (Fig.1A and B). Similarly, WT DCs induced the production of IL-2 from SM1 T cells within 6 h of culture, while TLR5-deficient DCs were unable to induce IL-2 production during this time period (Fig.1C). After 24 h of culture, TLR5-deficient DCs induced a small amount of IL-2, but this was still considerably less than detected in WT DC cultures (Fig.1C). Increased surface expression of CD69 and CD25 was also detected on SM1 T cells incubated with MyD88-deficient DCs, however, a delay in the kinetics of this response was noted when compared directly to WT DCs (Fig.1A and B). Similarly, IL-2 production from MyD88-deficient DC cultures was elevated when compared to TLR5-deficient DC cultures, but still low when compared to WT DCs (Fig.1C). These experiments confirm that TLR5 is required for robust activation of flagellin-specific T cells to whole flagellin. In contrast, MyD88 expression is not required for initial SM1 T-cell activation, but can contribute to the rapid kinetics of this response in vitro.

### Examination of protein phosphorylation after TLR5 ligation

**Figure 2:**
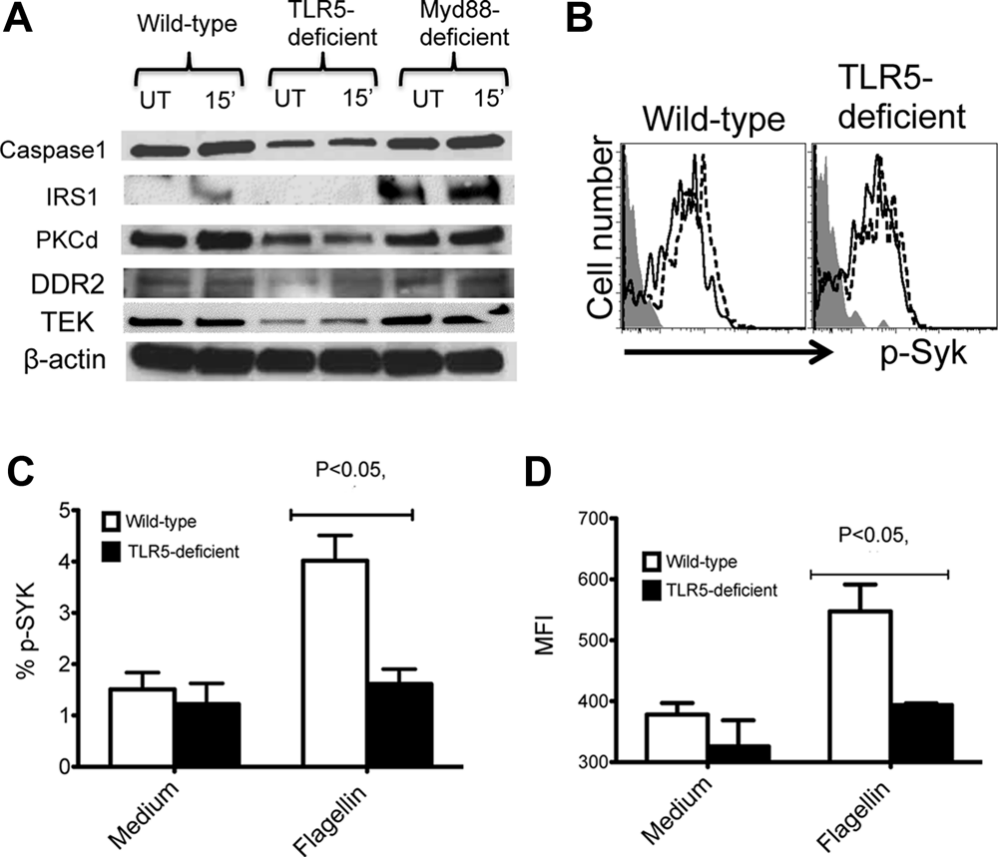
Flagellin induces phosphorylation and upregulation of molecules in DCs independently of MyD88. CD11c^+^ splenic DCs were enriched from WT, TLR5-deficient, and MyD88-deficient mice and treated with flagellin. (A) Whole cell lysates of CD11c^+^ DCs treated with flagellin (5 μg/mL) for 15 min and untreated control were analyzed by immunoblotting for caspase-1, IRS-1, PKC-d, DDR2, TEK, and b-actin (loading control). (B) Representative flow cytometry plots (*n* = 3 per group) show intracellular staining for Syk phosphorylation in WT and TLR5-deficient DCs treated with flagellin (10 ng/mL, dashed line histogram) or untreated (solid line histogram). Shaded histogram shows isotype control staining. (C and D) CD11c^+^ DCs from WT or TLR5-deficient mice were either treated with flagellin or left untreated. (C) The percentage and (D) MFI (mean fluorescence intensity) of phosphorylated Syk in CD11c^+^ DCs was determined by flow cytometry. Data are shown as mean + SEM of three samples per group, and are from a single experiment representative of two independent experiments. NS: nonsignificant by unpaired *t*-test.

TLR5 ligation induces the secretion of inflammatory mediators as a result of downstream signaling through MyD88, IRAK1/4, and other adaptors [8]. In order to identify other signaling molecules that might be engaged downstream of TLR5 in the absence of MyD88, we initially probed a Kinex antibody microarray containing a panel of phospho-specific antibodies to provide a broad overview of intracellular phosphorylation. Lysates were prepared from WT, TLR5-, and MyD88-deficient DCs activated in the presence of flagellin and assayed for protein phosphorylation. This screening approach highlighted several potential signaling molecules with increased phosphorylation in WT and MyD88-deficient DCs incubated with flagellin when compared to TLR5-deficient DCs (Table1). To confirm this initial screen, we used standard Western blotting to individually monitor phosphorylation changes in WT DC lysates after flagellin stimulation. However, using this conventional approach, we did not detect consistent or significant changes in the phosphorylation of these proteins in response to flagellin (Fig.2A). In contrast, we were able to detect a small amount of TLR5-dependent phosphorylation of Syk by flow cytometry after WT DCs were incubated with flagellin and importantly this response was consistently reduced in TLR5-deficient DCs (Fig.2B to D). These data suggested that flagellin might initiate Syk phosphorylation in a TLR5-dependent manner.

**Table 1:** Protein phosphorylation after flagellin stimulationa)

Protein phosphorylated	Estimated fold change,	
	Δ*f* = *F* (WT, Myd88) – *F* (TLR5)	
	WT	Myd88 deficient
PKCd	0.70	0.92
Tyro10 (DDR2)	0.66	0.77
Kit	1.12	0.74
CASP1	2.46	1.78
IRS1	1.13	0.96
GSK3b	2.50	3.74
TEK (TIE2)	1.40	1.20
Syk	5.51	5.28

a) DCs were isolated from WT, TLR5-deficient, and MyD88-deficient mice and stimulated with flagellin (5 μg/mL) for 15 min. Protein phosphorylation was determined in cell lysates with a Kinex antibody microarray, containing a panel of phospho-specific antibodies to provide a broad overview of intracellular phosphorylation. Estimated fold change (Δ*f*) is calculated by subtracting the fold change values observed in TLR5-deficient samples (control, F-TLR5) from WT (F-Wt) and MyD88-deficient (F-Myd88) samples. Fold change (*F*) is the *Z* ratio of treated over untreated samples.

### Inhibition of Syk reduces TLR5-dependent antigen presentation

**Figure 3:**
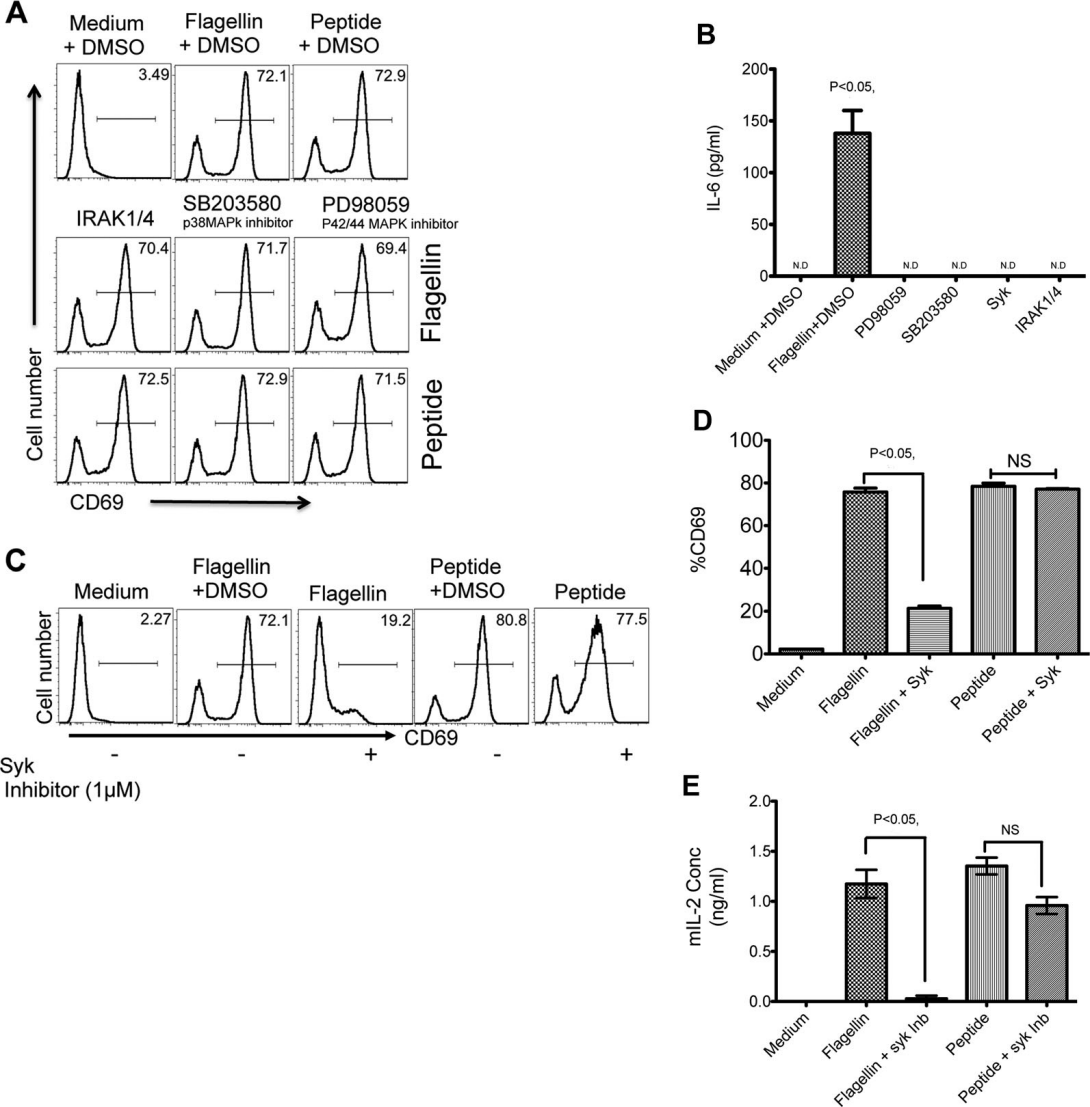
Syk is required for optimal activation of DCs and flagellin-specific T cells. (A) The role of IRAK1/4, p38, and p42/44 MAPK was examined by inhibition with 50 μM of specific pharmacological inhibitors IRAK1/4 inhibitor, p38 MAPK inhibitor (SB203580), and p42/44 MAPK inhibitor (PD98059). In total, 1 × 10^5^ CD11c^+^ DCs were treated with inhibitors or left untreated for 30 min before incubation with 1 × 10^5^ SM1 T cells, 10 ng/mL of flagellin, or 6 μM of flagellin peptide (427–441). SM1 (CD4^+^CD90.1^+^) T-cell activation was determined as CD69 expression by flow cytometry 16 h after flagellin stimulation. (B) IL-6 production was assayed in culture supernatants of DCs treated with various pharmacological inhibitors as mentioned above and cultured with SM1 T cells in a 1:1 ratio for 16 h by ELISA. (C) CD11c^+^ DCs were pretreated with 1 μM of Syk inhibitor (Bay IV 61–016) or DMSO before incubating with 1 × 10^5^ SM1 T cells flagellin or peptide stimulation. Representative flow cytometry plots (*n* = 3 samples per group) show CD69 expression on (CD4^+^CD90.1^+^) SM1 T cells 16 h after flagellin or peptide stimulation, as measured by flow cytometry. (D) The percentage of CD69 expression as measured in (C). (E) Production of IL-2 in culture supernatants was assessed by ELISA 16 h after incubation with medium, flagellin, peptide, in the presence or absence of Syk inhibitor. (B, D, and E) Data are shown as mean ± SEM of three samples per group, and are from one single experiment representative of two independent experiments. NS: nonsignificant by unpaired *t*-test.

As a complementary approach to direct examination of protein phosphorylation, we monitored the effect of inhibiting different signaling pathways during TLR5-dependent antigen presentation of flagellin to SM1 T cells. As expected, WT DCs were able to activate SM1 T cells in vitro to increase surface expression of CD69 and produce IL-2, when peptide or whole flagellin was added to cultures (Fig.3A, top row). The addition of IRAK1/4 (IRAK1/4 inhibitor), p38 MAP kinase (SB203580), or p42/44 mitogen-activated protein kinase (MAPK (PD98059) inhibitors to DCs had no discernable effect on their ability to induce CD69 expression on SM1 T cells incubated with whole flagellin or peptide (Fig.3A). However, each of these inhibitors was able to block IL-6 production in cultures, indicating that DC activation via TLR5 ligation was effectively inhibited (Fig.3B). In marked contrast, the addition of a Syk inhibitor (BAY 61–3606) completely blocked the ability of DCs to induce CD69 expression on SM1 T cells and IL-2 production in response to flagellin protein (Fig.3C to E). Importantly, the inhibition of Syk had no effect on SM1 T-cell stimulation in response to peptide (Fig.3C to E), demonstrating that Syk is only necessary when antigen processing is required.

### Syk and CARD9 are required for optimal antigen presentation to flagellin-specific T cells

**Figure 4:**
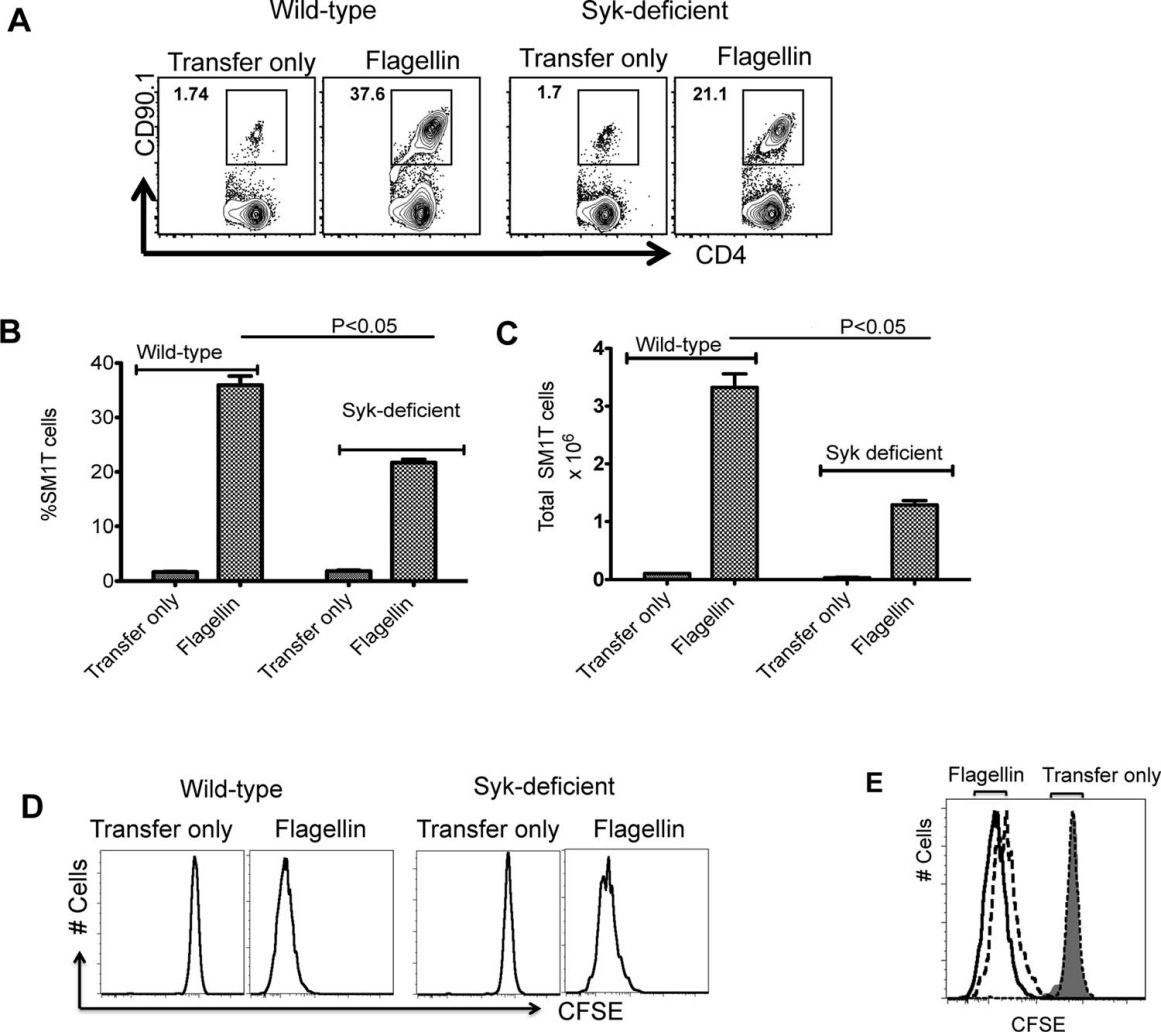
Syk deficiency hinders robust activation of SM1 T cells in vivo. (A) In total, 1 × 10^6^ CFSE-stained SM1 T cells were adoptively transferred into WT and Syk-deficient chimeric mice and the following day were immunized with 1 μg of flagellin. Three days later, clonal expansion of SM1 T cells was examined in the spleen by flow cytometry. Representative flow cytometry plots (*n* = 3 mice per group) are shown. (B and C) WT or Syk-deficient mice were immunized with flagellin (1 μg) and (B) the percentage and (C) total number of SM1 T cells in the spleens was determined by flow cytometry. Data are shown as mean + SEM of three mice per group, and are from a single experiment representative of three independent experiments. NS: nonsignificant by unpaired *t*-test. (D and E) SM1 T-cell proliferation in WT and Syk-deficient mice was examined by CFSE dye dilution. (E) WT (solid line), Syk-deficient mice (dotted line). Shaded area shows isotype control staining. Representative flow cytometry plots (*n* = 3 mice per group) are shown and are from one single experiment representative of three independent experiments.

Syk-deficient mice do not survive to full gestation, but a requirement for Syk in DC antigen presentation can be examined by using fetal liver chimeras [25]. We generated WT and Syk-deficient fetal liver chimeric mice and then tracked the in vivo response of SM1 T cells to flagellin immunization. Although SM1 T cells remained able to respond to flagellin immunization in Syk-deficient chimeras, CD4^+^ T-cell clonal expansion was significantly lower than observed in WT chimeras and there was a clear reduction in total SM1 T cells in the absence of Syk (Fig.4A to C). Furthermore, there was also a small reduction in CFSE dye-dilution by SM1 T cells stimulated in Syk-deficient versus WT chimeras (Fig.4D and E).

**Figure 5:**
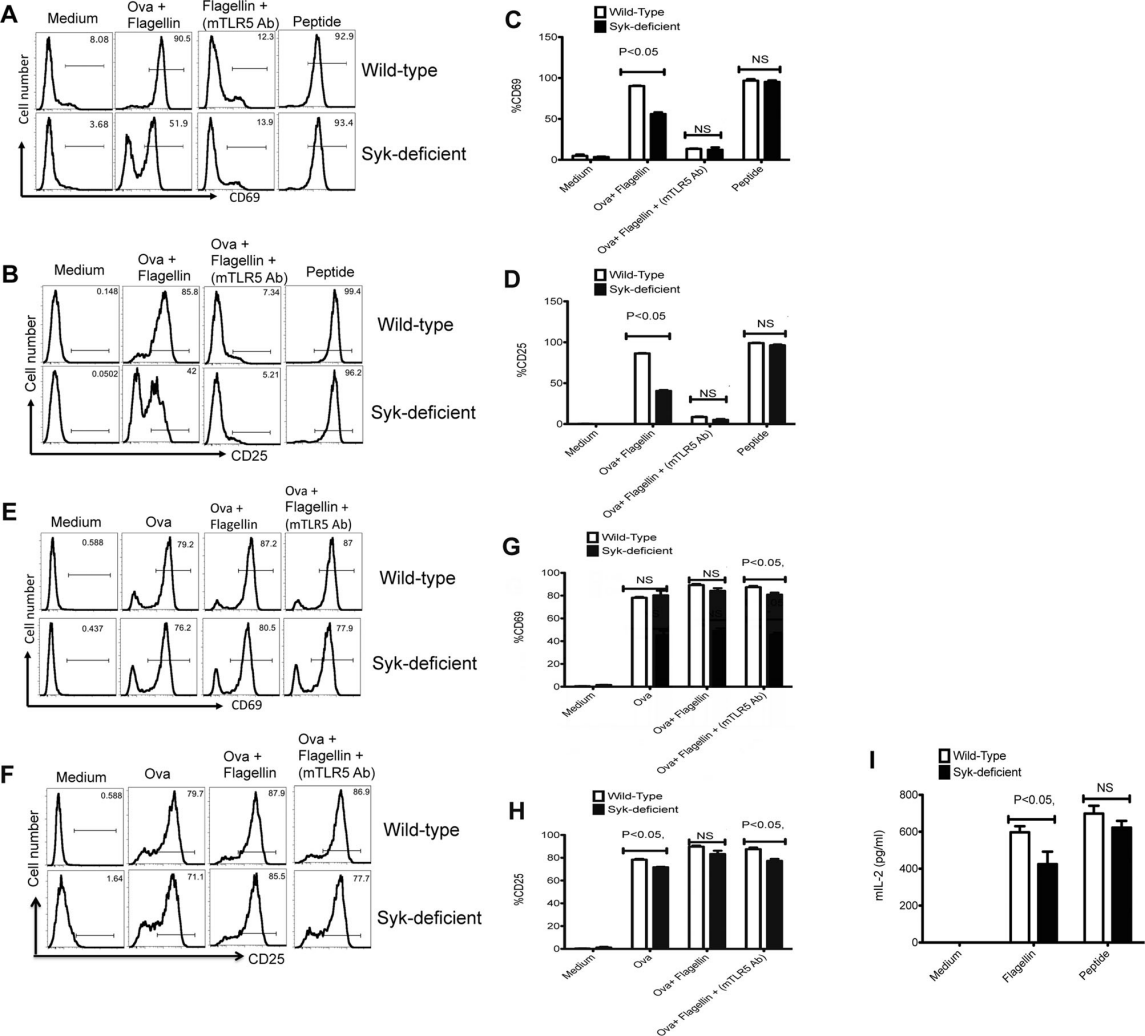
Syk-deficient DCs display a specific deficiency in the activation of flagellin specific CD4^+^ T cells. CD11c^+^ DCs (1 × 10^5^) isolated from WT and Syk-deficient chimeric mice were either pretreated with monoclonal anti-TLR5 antibody for 60 min or left untreated and were then cultured with 1 × 10^5^ SM1 or OT-II T cells for 16 h in the presence of OVA (100 μg/mL), OVA (100 μg/mL) + flagellin (1 ng/mL), or flagellin peptide (6 μM). (A and B) Representative flow cytometry plots (*n* = 3 samples per group) show the expression of (A) CD69 and (B) CD25 on (CD4^+^CD90.1^+^) SM1 T cells. (C and D) The percentage of (C) CD69- and (D) CD25-expressing SM1 T cells as treated in (A and B). (E and F) Representative flow cytometry plots (*n* = 3 samples per group) show the expression of (E) CD69 and (F) CD25 on (CD4^+^CD90.1^+^) OT-II T cells cultured with treated CD11c^+^ DCs enriched from spleen of WT and Syk-deficient chimeric mice as described above. (G and H) The percentage of (G) CD69- and (H) CD25-expressing OT-II T cells as treated in (E and H). (I) IL-2 concentration was measured in the culture supernatant of DC T-cell coculture experiments (16 h) by ELISA. (C, D, G, H, I) Data are shown as mean + SEM of three samples per group, and are from one experiment representative of three independent experiments. NS: nonsignificant by unpaired *t*-test.

Given the modest impact of Syk deficiency on SM1 T cells in vivo, it remained possible that some WT APCs transferred to chimeras during the T-cell adoptive transfer process were responsible for some of the T-cell response. To address this limitation, we directly examined the ability of enriched DCs from Syk-deficient chimeras to activate SM1 T cells in vitro. In order to have an internal control for these experiments, we simultaneously examined the ability of OT-II T cells to respond to OVA added to the same cultures. In these cultures, Syk-deficient DCs displayed a significantly lower ability than WT DCs in activating SM1 T cells to increase surface expression of CD69 or CD25 when flagellin protein was added to cultures (Fig.5A to D). In contrast, Syk-deficient DCs remained able to activate SM1 T cells when peptide was added to cultures (Fig.5A to D). Furthermore, the addition of an antibody specific for TLR5 was able to block antigen presentation of flagellin to SM1 T cells (Fig.5A to D), demonstrating that the antigen presentation in this culture is TLR5-dependent. In addition, cultures containing Syk-deficient DCs induced lower amounts of IL-2 production from SM1 T cells, when compared to WT DCs (Fig.5I). In these same cultures, both WT and Syk-deficient DCs were able to activate OVA-specific OT-II T cells to increase the expression of CD69 and CD25 (Fig.5E to H). Thus, the absence of Syk causes a specific deficiency in the ability of DCs to present flagellin to CD4^+^ T cells.

**Figure 6:**
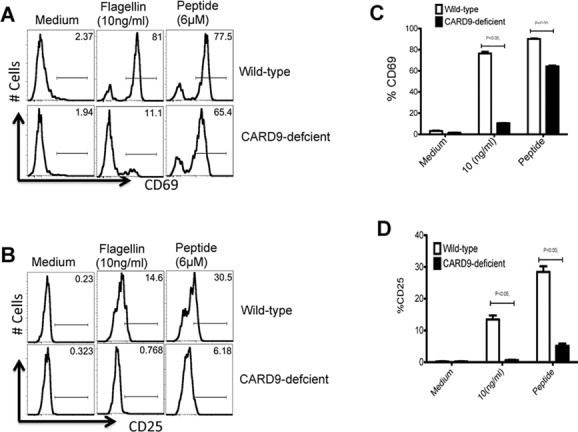
CARD9-deficient DCs display impaired ability to activate flagellin-specific T cells. CD11c^+^ DCs were enriched from the spleens of WT and CARD9-deficient mice. (A–D) In total, 1 × 10^5^ DCs were cultured with 1 × 10^5^ SM1 T cells in the presence of flagellin (10 ng/mL), peptide (6 μM), or medium alone for 16 h, and T-cell activation measured by flow cytometry. (A) T-cell activation was analyzed by measuring CD69 expression on gated SM1 T cells (CD4^+^CD90.1^+^) in the presence of WT (top) or CARD9-deficient (bottom) DCs. (C) The percentage of CD69^+^ SM1 T cells cultured with WT (white bars) or CARD9-deficient (black bars) DCs is shown. (B) T-cell activation was analyzed by measuring CD25 expression on gated SM1 T cells (CD4^+^CD90.1^+^) in the presence of WT or CARD9-deficient DCs. (D) The percentage of CD25^+^ SM1 T cells cultured with WT (white bars) or CARD9-deficient (black bars) DCs is shown. Histograms are representative of two individual experiments done in triplicate. (C and D) Data are shown as mean + SEM of three samples per group, and are from one experiment representative of two independent experiments. NS: nonsignificant by unpaired *t*-test.

CARD9 is a protein molecule that is downstream of the Dectin-1/Syk signaling pathway where it engages the Bcl-10-MALT1 complex to initiate transcription of genes involved in inflammation [26]. Given the requirement for Syk in the in vitro experiments above, we also decided to examine the role of CARD9 in DC activation of flagellin-specific T cells. When cultured with flagellin protein, CARD9-deficient DCs were completely unable to induce upregulation of CD69 and CD25 on SM1 T cells (Fig.6A to D). However, as observed with TLR5- and Syk-deficient DCs, the addition of processed flagellin peptide allowed both CARD9-deficient and WT DCs to efficiently activate SM1 T cells in vitro (Fig.6A to D). Interestingly, this effect of CARD9 deficiency appeared to be greater than the more modest effect of Syk deficiency, suggesting that other signaling pathways may remain able to activate CARD9 in the absence of Syk. Together, these data demonstrate that both Syk and CARD9 are required for optimal processing of whole flagellin for presentation to SM1 T cells.

## Discussion

Flagellin is an unusual molecule in that it is directly recognized by the receptors of the host innate and adaptive immune system [9–12]. Previous studies have demonstrated that TLR5-deficient mice have reduced antibody and T-cell responses to injected flagellin, but that a requirement for TLR5 is most clearly observed at low antigen concentrations [9–12]. Indeed, the sensitivity of mammalian adaptive immune responses to small quantities of flagellin initially led to the use of this antigen in many older immunological studies [6,22,23]. Our laboratory recently reported that host immune sensitivity to flagellin is mediated by TLR5 expression and that surprisingly this pathway displays limited dependence on MyD88, the only known adaptor protein used by TLR5. Therefore, our goal in this study was to examine whether other signaling pathways were required for the processing and presentation of flagellin protein to flagellin-specific CD4^+^ T cells.

**Figure 7:**
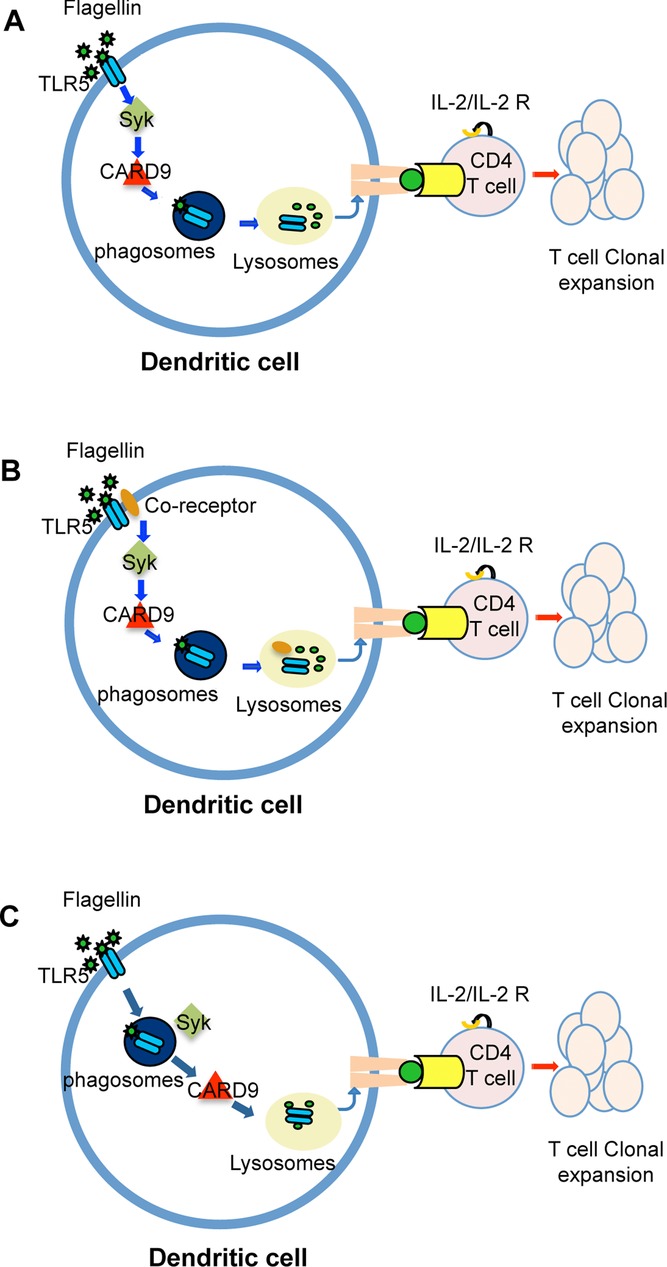
Proposed model for Syk and CARD9 involvement in TLR5-mediated flagellin antigen presentation. DCs expressing TLR5 can engage a noncanonical pathway via Syk and CARD9 to enhance flagellin specific T-cell activation. This may be the result of (A) direct signals via TLR5 to the Syk and CARD9 pathway to enhance flagellin processing and MHC class II loading during maturation of the phagosome/lysosome. This enhanced processing results in enhanced TCR ligation, upregulation of IL-2 production, and subsequent T-cell clonal expansion. (B, C) Alternatively, either (B) a Syk/CARD9 pathway may enhance flagellin processing downstream of a TLR5 coreceptor, or (C) Syk and CARD9 activation occurs during flagellin processing in the phago/lysosome, following Syk-independent, TLR5-mediated uptake of flagellin.

Unlike TLRs, CLR ligation initiates phosphorylation of Syk and resulting signals utilize CARD9 in a pathway that eventually leads to a proinflammatory response [13,26]. Specifically, in the absence of CARD9 or Syk, DCs were unable to robustly activate flagellin-specific T-cell responses. Thus, our data suggest that a similar pathway is operational during flagellin processing by DCs, although the exact intersection of Syk and CARD9 with the TLR5 pathway is currently unclear. Since the presentation of flagellin to T cells at low antigen doses is TLR5-dependent and an anti-TLR5 antibody blocked presentation in our assays, one possibility is that TLR5 signals downstream using a CLR-like pathway to enhance antigen presentation of bound ligands (Fig.7A). However, an alternative possibility is that a coreceptor is recruited following TLR5 ligation and specifically participates in the enhancement of flagellin uptake and antigen processing via Syk and CARD9 signaling pathways (Fig.7B). A final possibility is that TLR5 ligation initiates flagellin uptake independent of Syk and CARD9, but that the processing of flagellin epitopes requires Syk and CARD9 pathways (Fig.7C). The generation of new reagents to visualize flagellin/TLR5 trafficking in APCs will be required to discriminate between these different possibilities. It is of interest that the effect of Syk deficiency was considerably milder than that observed for CARD9 deficiency, perhaps indicating that there are other signaling pathways that can also converge on CARD9 in the absence of Syk. An involvement of Syk/CARD9 in flagellin antigen processing would be consistent with the known role of CLRs, which have been shown to utilize Syk-dependent pathways to enhance antigen presentation of bound ligands [27]. Given this similarity, it is tempting to speculate that any antigen that is conjugated to a TLR ligand may be able to preferentially enhance subsequent antigen-specific T-cell responses in a process that is fundamentally distinct from the well-described adjuvant effects of TLR ligands.

Flagellin can function as a potent adjuvant and DCs are activated within hours of injection of purified flagellin into naïve mice [28,29]. Depending on the dose or route of flagellin administration, this adjuvant effect can lead to the generation of Th1- or Th2-like adaptive responses and can require the expression of TLR5 or the cytoplasmic flagellin sensor NLRC4 [30–35]. However, the exquisite sensitivity of CD4^+^ T cells to low-dose flagellin administration is solely dependent on the expression of TLR5 [11,12]. Our experiments examining the early kinetics of T-cell activation have revealed that, although MyD88 is not required for flagellin-specific T-cell activation, it does play an important role in accelerating early T-cell activation, presumably as a result of previously described MyD88-dependent effects on DC costimulation and cytokine production [36]. Together these data suggest a model where flagellin ligation of TLR5 initiates a MyD88-dependent pathway that leads to DC activation and that this occurs simultaneously with a Syk-dependent pathway that encourages antigen presentation. The overall contribution of flagellin TLR5 binding to flagellin-specific T-cell activation is likely to be a result of both of these pathways working together.

Understanding the signaling pathways present downstream of TLRs is an important objective since they contribute to sepsis [37] and other inflammatory disorders [38]. TLRs have previously been shown to interact with CLR signaling during fungal recognition, where zymosan binds to both TLR and Dectin-1 and initiates both MyD88-dependent and MyD88-independent signaling pathways simultaneously [39]. It is clear that binding of flagellin to TLR5 initiates a signaling cascade through MyD88 that leads to the secretion of proinflammatory cytokines and recruitment of phagocytic cells to mediate pathogen clearance [40,41]. However, our studies here demonstrate that Syk-dependent signals are also initiated following TLR5 ligation and that MyD88 expression is not essential for antigen presentation that leads to flagellin-specific T-cell activation. This alternative-signaling pathway after TLR5 ligation has not previously been reported, but we speculate that this is because almost all previous studies have focused on innate inflammation. In contrast, our studies have specifically examined the contribution of TLR5 to the initiation of flagellin-specific adaptive immune responses. It is important to note that all of our DC and T-cell assays demonstrate no antigen-presentation deficiency when a flagellin peptide or OVA antigen presentation is examined [12], thus the contribution of the Syk-, CARD9-dependent pathway is only observed when examining CD4^+^ T cell responses to whole flagellin. While this may appear to be a relatively unique situation, this preferential treatment of flagellin may have specifically evolved to provide a rapid adaptive immune response to flagellated microbes and may explain the dominance of flagellin-specific responses noted in inflammatory bowel disease [42,43].

In conclusion, our study suggests the presence of a Syk- and CARD9-dependent pathway that operates in conjunction with TLR5 ligation to enhance flagellin antigen presentation. Future examination of this pathway could be important in the development of vaccines for flagellated microbes and understanding the initiation of flagellin-specific responses in inflammatory disease.

## Materials and methods

### Mice and reagents

C57BL/6 and B6.SJL-PtprcaPep3b/BoyJ (CD45.1 congenic) mice (6–8 weeks old) were purchased from The Jackson Laboratory (Bar Harbor, Maine, USA) and NCI (Frederick, MD). TLR5-deficient [44,45] and MyD88-deficient [46] mice were bred from breeder stock originally provided by Dr. A. Gewirtz (Georgia State University, GA) and Dr. S. Way (University of Minnesota Medical School, MN). Rag-deficient, CD90.1 congenic, flagellin-specific SM1 TCR transgenic mice were bred in our facility and have previously been described in detail [47,48]. Rag-deficient, OT-II TCR transgenic mice [49] were backcrossed to a Rag-deficient CD90.1 congenic background in our laboratory. CARD9-deficient [50] and Syk-deficient [25] mice were bred at the University of Texas MD Anderson Cancer Center and the MRC National Institute for Medical Research.

The flagellin epitope recognized by SM1 T cells (flagellin_427–441_) has previously been reported [51], and this peptide was purchased from Invitrogen Corporation (Carlsbad, CA). Ultrapure LPS was purchased from Alexis Biochemicals (Farmingdale, NY). Signaling pathway inhibitors SB203580 (4-(4-fluorophenyl)-2-(4-methylsulfinylphenyl)-5-(4-pyridyl)1H-imidazole), PD98059 (2′-amino-3′-ethoxyflavone), IRAK1/4 inhibitor (*N*-(2-morpholinylethyl)-2-(3-nitrobenzoylamido)-benzimidazole), and BAY 61–3606 (2-(7-(3,4-dimethoxyphenyl)-imidazo [1,2-c] pyrimidin-5-ylamino)-nicotinamide, HCl) were purchased from EMD Biosciences (Gibbstown, NJ).

### Fetal liver chimeric mice

WT and Syk-deficient chimeric mice were generated by IV injection of 1 × 10^6^ WT or Syk-deficient fetal liver cells [25] into B6.SJL-PtprcaPep3b/BoyJ (CD45.1 congenic) mice. Recipient mice were lethally irradiated with 2 × 600 rads using a ^137^Cs source before reconstitution. Reconstituted mice were treated with antibiotics for 8 weeks and the degree of chimerism (CD45.1 versus CD45.2) confirmed before use in in vitro and in vivo experiments.

### Bacterial strains and flagellin production

LPS-deficient *Salmonella* serovar Typhimurium X4700, provided by Dr. R. Curtiss (Arizona State University, Tempe, AZ), was used to purify flagellin using a modified acid-shock protocol. Overnight LB broth cultures were centrifuged, washed, and resuspended in PBS before acid treatment to liberate flagellin. Monomeric flagellin was prepared by depolymerizing dialyzed samples at 70°C for 1 h and passed through endotoxin removal columns (Pierce Biotechnology, Rockford, IL). Flagellin purity was determined using silver-stained SDS gels and endotoxin detection kits (Sigma, St. Louis, MO). Our flagellin preparations have previously been shown to have identical biological activity to recombinant flagellin produced in eukaryotic cells [10].

### Adoptive transfer and immunization

Single cell suspensions were prepared after harvesting spleen, inguinal, brachial, cervical, and mesenteric lymph nodes from SM1 TCR transgenic mice and red blood cells were lysed using ACK lysis buffer (Lonza, Walkersville, MD) before labeling with CFSE [52]. The frequency of SM1 T-cell was determined by flow cytometry before 0.8 – 1 × 10^6^ cells were transferred IV into recipient mice. The following day, mice were injected IV with flagellin and SM1 T cell expansion examined at various times afterward.

### DC isolation and in vitro stimulation

CD11c^+^ DCs were isolated from spleens digested using collagenase D (Roche Diagnostics, Indianapolis, IN), as previously described [10] and enriched to greater than 85–95% purity using CD11c microbeads (Miltenyi Biotech, Inc., Auburn, CA). Enriched DCs (1 × 10^5^ cell/well) were incubated in 96-well plates in a 1:1 ratio with SM1 or OT-II T cells in the presence or absence of added antigen. CD4^+^ T cells were recovered at 3, 6, 9, 12, 16, and 24 h time points after stimulation to examine T-cell activation and cytokine production. For inhibition assays, DCs were pretreated with inhibitors for 30 min and washed thoroughly before incubation with CD4^+^ T cells and added antigen. In some assays, the activity of TLR5 was blocked using 10 μg/mL of neutralizing anti-TLR5 monoclonal antibody (Invivogen) for 60 min before the addition of flagellin and SM1 T cells.

### Flow cytometric analysis

T-cell activation was examined using antibodies specific for CD4, CD90.1, CD69, and CD25 (eBiosciences). In some experiments, responding cells were permeabilized and stained with anti-phospho-Syk (Cell Signaling Technology, Inc., Danvers, MA) or isotype control antibodies. Cells were analyzed using a BD Fortessa and data analyzed using FlowJo software (Treestar, Inc., Ashland, OR).

### Cytokine ELISA

IL-2 and IL-6 cytokine production was examined in culture supernatants collected at various time points after culture (3, 6, 9, 12, and 24 h) using the mIL-2 BD Opt EIA detection kit (BD Biosciences, San Diego, CA) and mIL-6 according to manufacturer's protocol. Absorbance was measured at 450 nm using a microplate reader (Spectra Max M2, Molecular Devices, Inc., Sunnyvale, CA).

### Protein immunoblot analysis

Enriched splenic DCs were cultured in 96- or 24-well plates with purified flagellin (1 μg/mL or 10 ng/mL) and lysed at various time points with RIPA buffer (Sigma) containing protease inhibitors (Sigma). Whole-cell extracts were centrifuged, loaded onto 4–15% Tris/glycine gels (Bio-Rad, USA), and transferred to polyvinylidene fluoride or nitrocellulose membranes (PVDF)(GE Healthcare, Pittsburgh, PA, and Invitrogen). All antibodies (anti-phospho PKC-delta, anti-caspase-1, anti-β-actin, anti–TEK, and anti-phospho-IRS-1) were added at 1:1000 dilution, followed by HRP secondary antibodies (Jackson Immuno Research, West Grove, PA). ECL plus (GE Healthcare) was used as substrate and blots examined using a Kodak Image Station.

### Kinex antibody microarray preparation and data analysis

DCs were enriched from the spleens of WT, TLR5-deficient, and MyD88-deficient mice, using a CD11c^+^ DC isolation kit (Milteyni, Auburn). CD11c^+^ DCs (2 × 10^6^) from WT, TLR5-deficient, and MyD88-deficient were subsequently treated with flagellin (5 μg/mL) in culture for 15 min or left untreated. Stimulated and unstimulated cells were then washed in cold PBS and the lysates immediately prepared by sonication of for 5 min in Kinex lysis buffer (20 mM MOPS, 2 mM EGTA, 5 mM EDTA, 30 mM NaF, 1 mM Na_3_VO_4_, 1 mM PMSF, 5 μM pepstatin A, 1 mM DTT, 1% Triton X-100) supplemented with protease and phosphatase cocktail inhibitor. Samples were then centrifuged and approximately 100 μg of whole cell lysate from each sample were analyzed by Kinexus Bioinformatics Corporation (Vancouver, Canada) using the kinex antibody microarray service. For each phospho-antibody, Z ratios were calculated according to Kinex Antibody microarray protocols (Z ratio values of >1.0 is considered to be significant).

### Statistical analysis

Data were determined to be normally distributed and differences examined using InStat (GraphPad Software, La Jolla, CA). Data were compared using unpaired *t*-test and were considered significantly different with a *p* value of <0.05.

## Abbreviations

CLR: C-type lectin receptor
NLR: Nod-like receptor
Syk: spleen tyrosine kinase
