# Supplementary material for: Rapid CD4+ T-cell responses to bacterial flagellin require dendritic cell expression of Syk and CARD9
Source: Eur J Immunol. 2014 Dec 8;45(2):513–24. doi: 10.1002/eji.201444744 (PMC4324162; doi:10.1002/eji.201444744)
Supplement: Supplementary file 1 [file eji0045-0513-sd1.pdf]

Peer review correspondence

European Journal of Immunology

Rapid CD4^{+} T-cell responses to bacterial flagellin require dendritic cell expression of Syk and CARD9

Shaikh M. Atif, Seung-Joo Lee, Lin-Xi Li, Satoshi Uematsu, Shizuo Akira, Sara Gorjestani, Xin Lin, Edina Schweighoffer, Victor L. J. Tybulewicz and Stephen J. McSorley

Corresponding author: Stephen J. McSorley, Center for Comparative Medicine, Department of Anatomy, Physiology, and Cell Biology, UC Davis, Davis, CA

Review Timeline:	Submission date:	14 April 2014
	First editorial decision:	14 May 2014
	First revision received:	25 August 2014
	Second editorial decision:	26 September 2014
	Second revision received:	13 October 2014
	Accepted:	31 October 2014

Handling Executive Committee member: Prof. Caetano Reis e Sousa

Please note that the correspondence below does not include the standard editorial instructions regarding preparation and submission of revised manuscripts, only the scientific revisions requested and addressed.

First Editorial Decision – 14 May 2014

Dear Dr. McSorley,

Manuscript ID eji.201444744 entitled "Rapid CD4 T cell responses to bacterial flagellin requires dendritic cell Myd88-independent signals" which you submitted to the European Journal of Immunology has been reviewed. The comments of the referees are included at the bottom of this letter.

A revised version of your manuscript that takes into account the comments of the referees will be reconsidered for publication.

You should also pay close attention to the editorial comments included below. *In particular, please edit your figure legends to follow Journal standards as outlined in the editorial comments. Failure to do this will result in delays in the re-review process.*

European Journal of Immunology

Please note that submitting a revision of your manuscript does not guarantee eventual acceptance, and that your revision will be re-reviewed by the referees before a decision is rendered.

If the revision of the paper is expected to take more than three months, please inform the editorial office. Revisions taking longer than six months may be assessed by new referees to ensure the relevance and timeliness of the data.

Once again, thank you for submitting your manuscript to European Journal of Immunology and we look forward to receiving your revision.

Yours sincerely,
Karen Chu

On behalf of Prof. Bernard Malissen

Dr. Karen Chu
Editorial Office
European Journal of Immunology
e-mail: ejied@wiley.com
www.eji-journal.eu

****

Reviewer: 1

Comments to the Author
Although the authors conclude that a TLR5, but MyD88-independent pathway using Syk and CARD9 is involved in DC activation of antigen specific T cells, the data supporting this conclusion may have an alternative explanation that is quite different from that put forward by the authors.

1. Figure 1: The authors do not consider the possibility that the presence of TLR5 simply enhances the uptake of flagellin and thus facilitates its processing to peptide. The slow kinetics of response is certainly consistent with this conclusion. The authors need to conduct a kinetic analysis of flagellin breakdown and the appearance of the immunogenic peptide. The data in Figure 4 are consistent with this conclusion.
2. Figure 2: Why were so many different concentrations of flagellin used in the various experiments? In one experiment, the concentration is 5 micrograms/ml--an exceedingly high concentration. The overall quality of the data in this figure is extremely poor. The blots do not lend themselves to easy analysis. The authors need to quantitate the bands . The data in Figure 2C look like there was no significant effect on p-

European Journal of Immunology

Syk. The results in Figure 2D are plotted in a manner that makes small differences look large. The authors should avoid such an approach. Finally, because something is statistically signficiant, it does not mean it is physiological relevant. What data supports the conclusion that such small changes translate into major changes in cellular behavior/signaling?

3. Figure 3C: What is the evidence that the drugs were fully active? The authors need to conduct experiments to demonstrate specific inhibition of the involved enzymes.

4. Figure 4: As noted above, the data in this figure could also be used to conclude that TLR5 promotes efficient uptake and thus enhanced processing of flagellin as an antigen.

5. The authors should address the possibility that the Syk/CARD9 pathway contributes to the cleavage of flagellin.

Reviewer: 2

Comments to the Author
Previous articles by the authors showed that in vitro and in vivo the innate receptor Toll-Like Receptor 5 (TLR5) on dendritic cells promotes efficient presentation of its cognate ligand –the subunit protein of bacteria flagella or flagellin – to transgenic flagellin-specific CD4 T cells (SM1) (Letran et al., Eur. J. Immunol. 2011. 41: 29–38; Atif et al., Mucosal Immunology, 2014, vol7, number1; 68-77). This ability to facilitate presentation was shown to be independent of MYD88, the main transducer downstream of TLR family members. The present study is an important follow up that aims at deciphering the MYD88-independent pathway responsible for TLR-5-mediated flagellin presentation and proposes an involvement of Spleen Tyrosine Kinase (Syk) and CARD9 downstream of TLR5.

Major comments:
The authors show that in vitro, MYD88-independent stimulation of TLR5 by flagellin induces the phosphorylation of Syk. Further experiments in vitro show that blockade of Syk activity by chemical inhibitors or by genetically-engineered knockout impairs the ability of CD11c+ dendritic cells to activate SM1 cells upon flagellin exposure. Dendritic cells from mice deficient in CARD9, a known target of Syk, are also impaired for flagellin presentation to SM1 cells. Thus, this set of experiments in vitro points towards a novel pathway potentially important for TLR-5-mediated flagellin presentation to T cells, and involving Syk and CARD9, usually associated to c-type lectin signalling.

Next, the authors seek to validate their findings in vivo. They construct two groups of mouse bone marrow chimeras with respectively wild type or Syk-deficient immune cells, and in which they adoptively transfer SM1 CD4 T cells. Then, some of the chimeras from the two groups are immunized with flagellin, and compared for their ability to induce SM1 responses at day 3 after immunization. This experiment shows that clonal expansion of SM1 is significantly decreased in Syk-deficient compared to wild type chimeras.

European Journal of Immunology

However, there is still a substantial expansion of SM1, with SM1 cells representing 21,1% of the total CD4 T cells in Syk-deficient chimeras, versus 37.6% in WT chimeras. This phenotype seems much milder than that shown for TLR5-deficient mice in an article recently published by the authors (Figure 1B Atif et al., Mucosal Immunology, 2014, vol7,number1; 68-77). Therefore, it is still unclear from this analysis how this molecular pathway actually contributes to TLR5-mediated flagellin presentation in vivo, and this important point requires experimental clarification.

The reduction in absolute numbers of SM1 cells in Syk-deficient chimeras (Figure 4C) could reflect delayed T cell activation / proliferation as proposed in the title of the manuscript. In this case, clonal expansion in Syk-/- chimeras should catch up and reach that observed in WT mice at later time points. Because there is no kinetics study of SM1 activation in vivo, this conclusion cannot be reached. Alternatively, Syk deficiency could lead to more severely impaired T cell responses with failure to produce effector T cells (i.e, cytokine producers).

To answer these questions, the authors should:

1. show the exact level of in vivo proliferation of SM1 in WT or Syk-deficient chimeras, as assessed by CFSE dilution at day 3 after flagellin immunization, and the levels of expression of activation markers (CD69, CD25, D62L, CD44). This would indicate if the diminution of SM1 expansion is due to abortive proliferation/activation, and/or if some SM1 cells undergo full differentiation program into effector cells.

2. assess the ability of the responding SM1 to produce IL-2 (as in the in vitro assay Figure 3D of this manuscript), and differentiate into effector cells for instance by testing IFN-g and IL-4 production (McSorley. J Immunol 2002; 169:3914-3919, Didierlaurent et al., J Immunol ; 172: 6922–6930 in this same experimental setup.

Without this supplement of information, the role of TLR5/Syk during T cell responses to flagellin in vivo still remains elusive.

Finally, the results should be strengthen by providing statistical significance to highlight differences between experimental groups (Figures 1; 2D and E: in 2E what does ** stand for?; 3 and 5), the number of repeats (Figures 1, 2, 4) and the number of mice involved in the experiments (Figure 4A-B).

Minor points:
1/ Title:
The authors have shown previously that MYD88 is dispensable to mediate the effect of TLR-5 for presentation to CD4 T cells (Letran et al., Eur. J. Immunol. 2011. 41: 29–38; Atif et al., Mucosal

European Journal of Immunology

Immunology, 2014, vol7, number1; 68-77), it is surprising that the title at present does not reflect better the novelty of the finding involving Syk downstream of TLR5.

2/ Introduction

Page 4 lines 29/30 “ASC” should be written in full or referred to as “adaptor protein ASC” to avoid confusion with different entities sharing the same acronym.

3/ Material and Methods:

A/ The source of anti-TEK is not indicated

B/ Some reagents or procedures are listed for which no data are presented; the corresponding data should be shown

- CFSE staining (page 15 line42)
- study of the activation of SM1 cells in bone marrow chimeras at various time points after immunization (page 15 line 47)
- antibodies against CD8a, CD11b, CD44 and CD62L (page 16 line 25-29)

4/ In the Figures:

Readability of the figures should be improved, in particular by:

A) setting threshold in FACS histograms and showing percentages with standard deviation and significance (Figures 3A, C; 4D-G, 5A-B)

B) Figure 2B: the authors should show a quantification of the signals obtained by western blot. It seems that there is some induction of Syk phosphorylation in TLR5-/- at 15 min, which is not consistent with the results obtained in Figure 2C with the anti-pSyk antibody. How does this induction on western blot compare with the results in table 1 from the arrays? This point should be discussed in Results section with the support of the quantification of the western blot.

C) Figure 2C should present a single, bigger-sized overlay histogram showing levels of p-Syk in wild type and TLR5-deficient cells. As it stands, differences in the degree of Syk phosphorylation are not obvious.

D) Increasing the size of text and numbers, for the current size is not visible on printout, and requires maximum magnification on computer screen, especially Figure 4.

5/ Discussion: sentence page 12 line 12 should be edited. “However, ..." “...cytokine production”

European Journal of Immunology

Reviewer: 3

Comments to the Author

The manuscript “Rapid CD4 T cell responses to bacterial flagellin requires dendritic cell Myd88-independent signals” by Atif et al. is a logical progression of their very nice studies that investigate recognition of bacterial flagellin and the generation of CD4 T cell immunity. The paper shows new evidence that Syk and CARD9 signaling are downstream of TLR5, and responsible for enhancing the TLR5-dependent, MyD88-independent CD4 T cell responses to flagellin. The paper has the potential to make important contributions to our understanding of flagellin based recognition by the immune system and signaling through innate immune receptors. There are a few issues outlined below that if addressed will strengthen the authors data and conclusions.

1. In figure 2, the phospho-Syk western blot data are a little more convincing than the flow cytometry data, but the quality of both data make it difficult to interpret. It is not clear why the 15 min time point was chosen. Any statistical analysis for blot data? In addition, different doses of flagellin are used in each panel, ranging from 5 ug/ml to 10 ng/ml, and not explanation is given for this. Do the findings in “a” hold for lower, more physiologically relevant concentrations? In addition, in “d” the bar graph indicates there is no Syk phosphorylation above background in the TLR5-/- DC, whereas the blot in “b” looks like there is clear phosphorylation above background. There is also no explanation for how %Syk phosphorylation is calculated in “d” – what was used to normalize the signal between blots? For “e” MFI is shown, which does not take into account changes in background staining between cell preparations and experiments – change in MFI over background would be more appropriate since these are paired observations.
2. How do you know the peptide control is really relevant? Is the antigen density seen with peptide equal to what is achieved with 1 ng/ml flagellin? Since higher doses of flagellin are TLR5-independent, it would seem that the peptide dose may also be relevant. Has the peptide dose been titrated to show that there is no difference between WT and TLR5-/- DC?
3. Same is true for the ovalbumin control. If the phenotype is only true for very low doses of flagellin, then dose responses with controls seem justified. The molar ratios of both the peptide and ovalbumin are substantially higher than the amount of flagellin used. You would get the same data as you do for ovalbumin, if you used a higher concentration of flagellin. Your conclusions would be strengthened by showing dose response data for controls, in order to demonstrate that the TLR5 phenotype is flagellin specific.

European Journal of Immunology

First revision – authors' response – 25 August 2014

Reviewer: 1

Although the authors conclude that a TLR5, but MyD88-independent pathway using Syk and CARD9 is involved in DC activation of antigen specific T cells, the data supporting this conclusion may have an alternative explanation that is quite different from that put forward by the authors.

1. Figure 1: The authors do not consider the possibility that the presence of TLR5 simply enhances the uptake of flagellin and thus facilitates its processing to peptide. The slow kinetics of response is certainly consistent with this conclusion. The authors need to conduct a kinetic analysis of flagellin breakdown and the appearance of the immunogenic peptide. The data in Figure 4 are consistent with this conclusion.

We agree that this hypothesis would be consistent with our data. However, we would point out that simply enhancing uptake of flagellin would still require the involvement of Syk and CARD9. Unfortunately, in the absence of new reagents (either flagellin, TLR5, or MHC-peptide-specific antibodies) we have no way of examining the intracellular uptake or processing of flagellin within DCs, or the appearance of immunogenic peptide on the surface. Currently, the only assay that we have that allows us to track flagellin processing or presentation is the functional assay that we use extensively in our manuscript, i.e. the activation of flagellin-specific CD4 T cells. Thus, while we agree that examining flagellin breakdown in APCs would improve our manuscript, we are technically unable to address this issue at present. In response to the reviewer's comments, we have incorporated a discussion of the possibility of enhanced flagellin uptake and processing in the discussion section of the revised manuscript.

2. Figure 2: Why were so many different concentrations of flagellin used in the various experiments? In one experiment, the concentration is 5 micrograms/ml--an exceedingly high concentration. The overall quality of the data in this figure is extremely poor. The blots do not lend themselves to easy analysis. The authors need to quantitate the bands. The data in Figure 2C look like there was no significant effect on p-Syk. The results in Figure 2D are plotted in a manner that makes small differences look large. The authors should avoid such an approach. Finally, because something is statistically signficiant, it does not mean it is physiological relevant. What data supports the conclusion that such small changes translate into major changes in cellular behavior/signaling?

We agree with the reviewer that the immunoblot data are of fairly low quality and are difficult to interpret. We have made several attempts to generate higher quality immunoblot data, however, when examining primary cells these antibodies are only capable of detecting small phosphorylation changes with considerable background that means the data are at best suggestive. Given similar comments below by other Reviewers we have decided to remove phospho-Syk blotting data (prior Fig. 2B) from the revised manuscript. However, we would point out that we have been able to confirm a role for Syk using several different experimental approaches (flow cytometry, chemical inhibitors, and gene-deficient chimeras) we therefore feel that our overall conclusion that Syk signaling is required for flagellin DC presentation remains solid.

European Journal of Immunology

3. Figure 3C: What is the evidence that the drugs were fully active? The authors need to conduct experiments to demonstrate specific inhibition of the involved enzymes.

The reviewer may have missed our data showing that each of these inhibitors blocks the production of IL-6 when compared to vehicle controls (Fig. 3B). Thus, each of the drugs used in our experiments has been verified to be active and the absence of an effect on flagellin processing is not due to any inactivity at the concentrations used.

4. Figure 4: As noted above, the data in this figure could also be used to conclude that TLR5 promotes efficient uptake and thus enhanced processing of flagellin as an antigen.

We agree. However, as noted above, there are currently no reagents that allow direct examination of the breakdown of flagellin inside antigen presenting cells and thus we are unable to confirm this experimentally. It should be noted that while several studies have reported TLR5 surface staining using antibodies, our laboratory has examined every available TLR5 antibody including one that was recently described to display clear staining potential by western and flow cytometry (Shibata et al, 2012, Int. Immunol. 24:613). While some of these antibodies block functional effects of flagellin binding to TLR5, in our hands, none of them can reliably stain TLR5 on any murine cell type when rigorous controls are used. Furthermore, the most widely used flagellin-specific monoclonal antibody for examining flagellin by western blot is now no longer available from suppliers. Thus, it is currently not possible to track either TLR5 or flagellin breakdown inside cells.

5. The authors should address the possibility that the Syk/CARD9 pathway contributes to the cleavage of flagellin.

We agree with the reviewer that flagellin processing may be tightly linked to the involvement of the Syk/CARD9 pathway and have now incorporated this possibility into our discussion.

Reviewer: 2

Previous articles by the authors showed that in vitro and in vivo the innate receptor Toll-Like Receptor 5 (TLR5) on dendritic cells promotes efficient presentation of its cognate ligand –the subunit protein of bacteria flagella or flagellin – to transgenic flagellin-specific CD4 T cells (SM1) (Letran et al., Eur. J. Immunol. 2011. 41: 29–38; Atif et al., Mucosal Immunology, 2014, vol7, number1; 68-77). This ability to facilitate presentation was shown to be independent of MYD88, the main transducer downstream of TLR family members. The present study is an important follow up that aims at deciphering the MYD88-independent pathway responsible for TLR-5-mediated flagellin presentation and proposes an involvement of Spleen Tyrosine Kinase (Syk) and CARD9 downstream of TLR5.

European Journal of Immunology

Major comments:

The authors show that in vitro, MYD88-independent stimulation of TLR5 by flagellin induces the phosphorylation of Syk. Further experiments in vitro show that blockade of Syk activity by chemical inhibitors or by genetically-engineered knockout impairs the ability of CD11c+ dendritic cells to activate SM1 cells upon flagellin exposure. Dendritic cells from mice deficient in CARD9, a known target of Syk, are also impaired for flagellin presentation to SM1 cells. Thus, this set of experiments in vitro points towards a novel pathway potentially important for TLR-5-mediated flagellin presentation to T cells, and involving Syk and CARD9, usually associated to c-type lectin signalling.

Next, the authors seek to validate their findings in vivo. They construct two groups of mouse bone marrow chimeras with respectively wild type or Syk-deficient immune cells, and in which they adoptively transfer SM1 CD4 T cells. Then, some of the chimeras from the two groups are immunized with flagellin, and compared for their ability to induce SM1 responses at day 3 after immunization. This experiment shows that clonal expansion of SM1 is significantly decreased in Syk-deficient compared to wild type chimeras. However, there is still a substantial expansion of SM1, with SM1 cells representing 21,1% of the total CD4 T cells in Syk-deficient chimeras, versus 37.6% in WT chimeras. This phenotype seems much milder than that shown for TLR5-deficient mice in an article recently published by the authors (Figure 1B Atif et al., Mucosal Immunology, 2014, vol7,number1; 68-77). Therefore, it is still unclear from this analysis how this molecular pathway actually contributes to TLR5-mediated flagellin presentation in vivo, and this important point requires experimental clarification.

We thank the reviewer for this positive assessment of our manuscript. We agree that the phenotype of the Syk-deficient chimeras is unexpectedly milder than TLR5-, and even the CARD9-deficient mice also described in our manuscript. It is not yet clear why this is the case but perhaps suggests a redundant role for Syk and other unknown signaling molecules downstream of TLR5 and upstream of CARD9. This possibility is now discussed in the revised manuscript.

The reduction in absolute numbers of SM1 cells in Syk-deficient chimeras (Figure 4C) could reflect delayed T cell activation / proliferation as proposed in the title of the manuscript. In this case, clonal expansion in Syk-/- chimeras should catch up and reach that observed in WT mice at later time points. Because there is no kinetics study of SM1 activation in vivo, this conclusion cannot be reached. Alternatively, Syk deficiency could lead to more severely impaired T cell responses with failure to produce effector T cells (i.e, cytokine producers).

To answer these questions, the authors should:

1. show the exact level of in vivo proliferation of SM1 in WT or Syk-deficient chimeras, as assessed by CFSE dilution at day 3 after flagellin immunization, and the levels of expression of activation markers (CD69, CD25, D62L, CD44). This would indicate if the diminution of SM1 expansion is due to abortive proliferation/activation, and/or if some SM1 cells undergo full differentiation program into effector cells.

European Journal of Immunology

In response to the reviewer we have added new data showing CFSE dye dilution comparing SM1 T cells and Wt and Syk-deficient chimeras (Revised Fig. 4D and E). These data show a slightly lower rate of CFSE dye dilution SM1 cells activated in Syk-deficient chimeras, supporting the idea of a deficiency in initial activation. Unfortunately, we did not generate any data examining activation marker expression from these same experiments. This new data has been discussed in the revised manuscript.

2. assess the ability of the responding SM1 to produce IL-2 (as in the in vitro assay Figure 3D of this manuscript), and differentiate into effector cells for instance by testing IFN-g and IL-4 production (McSorley. J Immunol 2002; 169:3914-3919, Didierlaurent et al., J Immunol ; 172: 6922–6930 in this same experimental setup.

Given the short period of in vitro stimulation, no IL-4 or IFN-g is detected after in vitro stimulation of SM1 T cells. As requested, we have now added new data showing the production of IL-2 following stimulation of SM1 T cells using Wt or Syk-deficient APCs (Revised Fig. 5I). These data do show significantly lower production of IL-2 when SM1 T cells are stimulated in the presence of Syk-deficient DCs. These new data are discussed in the revised manuscript.

Without this supplement of information, the role of TLR5/Syk during T cell responses to flagellin in vivo still remains elusive.

Finally, the results should be strengthen by providing statistical significance to highlight differences between experimental groups (Figures 1; 2D and E: in 2E what does ** stand for?; 3 and 5), the number of repeats (Figures 1, 2, 4) and the number of mice involved in the experiments (Figure 4A-B).

As requested, Figure 1 now shows statistical analysis of T cell activation and highlights differences between the groups. In Figure 2, ** stands for $p<0.05$ and this is now stated clearly. Figures 3, 5 and 6 now contain gates and bar graphs for histograms and the statistical significance of differences between experimental groups. The number of mice and repeat experiments has also been added to figure legends.

Minor points:

1/ Title:

The authors have shown previously that MYD88 is dispensable to mediate the effect of TLR-5 for presentation to CD4 T cells (Letran et al., Eur. J. Immunol. 2011. 41: 29–38; Atif et al., Mucosal Immunology, 2014, vol7, number1; 68-77), it is surprising that the title at present does not reflect better the novelty of the finding involving Syk downstream of TLR5.

As suggested by the reviewer, we have now modified the title to better reflect the data presented in the manuscript.

European Journal of Immunology

2/ Introduction

Page 4 lines 29/30 "ASC" should be written in full or referred to as "adaptor protein ASC" to avoid confusion with different entities sharing the same acronym.

This has now been modified in the text.

3/ Material and Methods:

A/ The source of anti-TEK is not indicated

B/ Some reagents or procedures are listed for which no data are presented; the corresponding data should be shown

- CFSE staining (page 15 line42)

- study of the activation of SM1 cells in bone marrow chimeras at various time points after immunization (page 15 line 47)

- antibodies against CD8a, CD11b, CD44 and CD62L (page 16 line 25-29)

We apologize for this oversight. The Materials and methods have now been revised and updated to reflect only the data that is actually presented in the paper.

4/ In the Figures:

Readability of the figures should be improved, in particular by:

A) setting threshold in FACS histograms and showing percentages with standard deviation and significance (Figures 3A, C; 4D-G, 5A-B)

As suggested by the Reviewer, Figures 3, 5 and 6 (prior Figures 4 and 5) have now been modified to include gates for histograms and bar graphs with the means and SD for each group. Because this change caused a large increase in the number of panels in Figure 4, we decided to spread this data between two modified Figures that show the in vitro (new Fig. 4) and in vivo (new Fig. 5) results using Syk-deficient chimeras.

B) Figure 2B: the authors should show a quantification of the signals obtained by western blot. It seems that there is some induction of Syk phosphorylation in TLR5-/- at 15 min, which is not consistent with the

European Journal of Immunology

results obtained in Figure 2C with the anti-pSyk antibody. How does this induction on western blot compare with the results in table 1 from the arrays? This point should be discussed in Results section with the support of the quantification of the western blot.

Given the criticism of the quality of the Syk phosphor-blot data by Reviewers, we have decided to remove Fig. 2B from the revised manuscript.

C) Figure 2C should present a single, bigger-sized overlay histogram showing levels of p-Syk in wild type and TLR5-deficient cells. As it stands, differences in the degree of Syk phosphorylation are not obvious.

In response to the reviewer, we have enlarged Fig. 2C (now revised Fig. 2B) in the revised manuscript.

D) Increasing the size of text and numbers, for the current size is not visible on printout, and requires maximum magnification on computer screen, especially Figure 4.

The size of text in Figure 4 was very small due the large number of individual panels in one figure. As noted above, given the reviewer's request to add mean and SD data for Fig. 4D-G, the revised manuscript has new figures showing in vivo (revised Fig. 4) and in vitro data (revised Fig. 5) seperately. Hopefully, this has improved the overall visibility of the individual panels.

5/ Discussion: sentence page 12 line 12 should be edited. “However, ..." “...cytokine production”

This sentence has been edited as suggested.

Reviewer: 3

Comments to the Author

The manuscript “Rapid CD4 T cell responses to bacterial flagellin requires dendritic cell Myd88-independent signals” by Atif et al. is a logical progression of their very nice studies that investigate recognition of bacterial flagellin and the generation of CD4 T cell immunity. The paper shows new evidence that Syk and CARD9 signaling are downstream of TLR5, and responsible for enhancing the TLR5-dependent, MyD88-independent CD4 T cell responses to flagellin. The paper has the potential to make important contributions to our understanding of flagellin based recognition by the immune system and signaling through innate immune receptors. There are a few issues outlined below that if addressed will strengthen the authors data and conclusions.

European Journal of Immunology

We would like to thank the reviewer for this positive assessment of the manuscript.

1. In figure 2, the phospho-Syk western blot data are a little more convincing than the flow cytometry data, but the quality of both data make it difficult to interpret. It is not clear why the 15 min time point was chosen. Any statistical analysis for blot data? In addition, different doses of flagellin are used in each panel, ranging from 5 ug/ml to 10 ng/ml, and not explanation is given for this. Do the findings in “a” hold for lower, more physiologically relevant concentrations? In addition, in “d” the bar graph indicates there is no Syk phosphorylation above background in the TLR5-/- DC, whereas the blot in “b” looks like there is clear phosphorylation above background. There is also no explanation for how %Syk phosphorylation is calculated in “d” – what was used to normalize the signal between blots? For “e” MFI is shown, which does not take into account changes in background staining between cell preparations and experiments – change in MFI over background would be more appropriate since these are paired observations.

Given the overall comments from the three reviewers, we decided to remove the in vitro Syk phosphorylation blot data from the revised manuscript. The remaining data still show Syk phosphorylation by flow cytometry, the functional effects of Syk inhibition and gene-deficiency. The data in Figure 2D show phosph-Syk staining for cells incubated for medium alone (background staining) and there is no difference between Wt and TLR5-deficient groups. Our preference is to show all the data including the medium alone control rather than simply subtract this signal from the positive staining observed in the presence of flagellin, since readers may then question whether any differences observed are actually due to significant differences in the baseline value for these cells.

2. How do you know the peptide control is really relevant? Is the antigen density seen with peptide equal to what is achieved with 1 ng/ml flagellin? Since higher doses of flagellin are TLR5-independent, it would seem that the peptide dose may also be relevant. Has the peptide dose been titrated to show that there is no difference between WT and TLR5-/- DC?

3. Same is true for the ovalbumin control. If the phenotype is only true for very low doses of flagellin, then dose responses with controls seem justified. The molar ratios of both the peptide and ovalbumin are substantially higher than the amount of flagellin used. You would get the same data as you do for ovalbumin, if you used a higher concentration of flagellin. Your conclusions would be strengthened by showing dose response data for controls, in order to demonstrate that the TLR5 phenotype is flagellin specific.

We agree that this is a very important question. In fact we addressed this issue directly in our previous publication (Atif, SM et al, 2014, Mucosal Immunol. 7:68) but neglected to mention this in the manuscript. In the previous paper, we examined the ability of Wt or TLR5-deficient DCs to stimulate flagellin-specific and OVA-specific T cells after stimulation with a range of doses of whole OVA or flagellin peptide Crucially, these published data show no difference in the ability of Wt and TLR5-deficient DCs to stimulate SM1 T cells or OVA-specific T cells across a wide range of doses of OVA or flagellin peptide. Only when flagellin protein is titrated, do we detect a deficiency in antigen presentation from TLR5-deficient DCs.

European Journal of Immunology

Thus, the effect that we observe is specific to TLR5-deficient DCs and specific to flagellin protein. We now highlight these important published data in more detail in the revised manuscript.

Second Editorial Decision – 26 September 2014

Dear Dr. McSorley,

Please accept my sincere apologies for the prolonged delay in processing the re-review of your manuscript ID eji.201444744.R1 entitled "Rapid CD4 T cell responses to bacterial flagellin require dendritic cell expression of Syk and CARD9", submitted to the European Journal of Immunology. There was a severely delayed report and a difference in opinion for which we sought additional advice.

Your manuscript has been re-reviewed and the comments of the referees are included at the bottom of this letter. The referee #1 was not satisfied with the revisions made and suggested rejection. We therefore asked for another opinion. The Executive editor has re-assessed the manuscript and agrees with the reviewer #1 that there are alternative explanations for the data (See comments below). The journal does not encourage multiple rounds of revision, but we would consider for publication a revised version of the manuscript that responds ref#1 and Executive editor's concerns, and highlights the alternatives indicated. You should fully address the concerns of the referee and the Executive editor in this final round of revision.

You should also pay close attention to the editorial comments included below.

Please note that submitting a revision of your manuscript does not guarantee eventual acceptance, and that your revision will be re-reviewed by the referee(s) before a decision is rendered.

If the revision of the paper is expected to take more than three months, please inform the editorial office. Revisions taking longer than six months may be assessed by new referee(s) to ensure the relevance and timeliness of the data.

Once again, thank you for submitting your manuscript to European Journal of Immunology and we look forward to receiving your revision.

Yours sincerely,
Laura Soto Vazquez

On behalf of Prof. Bernard Malissen

European Journal of Immunology

Editorial Office
European Journal of Immunology
e-mail: ejied@wiley.com
www.eji-journal.eu

****

Executive editor

I think Ref#1 is absolutely correct. I have grave concerns about the interpretation. The problem, in my view, is that there are zero data in the manuscript indicating that Syk/CARD9 are downstream of TLR5. Ref#1 line of reasoning is that everything could be explained by TLR5-mediated flagellin uptake followed by handover to a Syk/CARD9 coupled receptor that regulates antigen processing. Along the same lines, I feel that the data could also mean that there is a TLR5 co-receptor that signals via Syk, as shown for other TLRs (for example, CD14 and TLR4), and that antigen processing requires synergistic signalling by CLR/Syk and TLR/MyD88 pathways (which is extremely potent - see Eur J Immunol 38, 500–506 (2008)). The reason for suspecting synergy is that If you look at Fig. 1, contrary to the authors' assertion, there is a massive reduction of response when using MyD88-deficient DCs, greater than the deficiency in Fig. 5 when using Syk-deficient DCs. The reason for stating it is a co-receptor is that, of course, it is not there in TLR5-deficient cells (or, at least, when alone cannot signal in response to flagellin). So, in my view, the model in Fig. 7 is by no means proven and the whole message of the paper may be incorrect.

Other issues brought up during review are also important: Fig. 2 is absolutely not convincing and the quantitation of pSyk by FACS makes no sense (% positive cells? the staining, if real, looks unimodal).

The data are interesting and, if written appropriately, the paper would be a useful contribution.

Reviewer: 1

Comments to the Author
The authors have not addressed my concerns. Their comment that the reagents required to assess flagellin uptake and degradation are lacking are not valid.
The authors could:
1) obtain radiolabeled protein from bacterial cultures;
2) iodinate the protein;
or 3) tag the protein with a fluorescent label, e.g., an AlexFluor dye.

Without the requested data, the authors' conclusions are simply not well-founded.

Reviewer: 2

Comments to the Author

The data presented in the revised manuscript support the conclusions claimed by the authors and have been strengthened by new data and statistics. The contribution and significance of the findings are objectively discussed. Their novelties justify a publication in EJI as they will lead to new ways to modulate antigen presentation of a widely represented bacterial component - i.e. flagellin - towards different applications in the context of vaccine design or to dampen chronic inflammations.

Minor points:

1/ There is a problem in figure 5 G that is an overlay of two of the same bar chart.

2/ The same p value ($p<0.05$) is not associated consistently with the same symbols, sometimes *, ** or ***. These symbols could be removed as the p value is always specified on the figures.

Reviewer: 3

Comments to the Author

The authors have adequately addressed all of my concerns.

<u>Second revision – authors' response – 13 October 2014</u>

Executive editor

I think Ref#1 is absolutely correct. I have grave concerns about the interpretation. The problem, in my view, is that there are zero data in the manuscript indicating that Syk/CARD9 are downstream of TLR5. Ref#1 line of reasoning is that everything could be explained by TLR5-mediated flagellin uptake followed by handover to a Syk/CARD9 coupled receptor that regulates antigen processing. Along the same lines, I feel that the data could also mean that there is a TLR5 co-receptor that signals via Syk, as shown for other TLRs (for example, CD14 and TLR4), and that antigen processing requires synergistic signalling by CLR/Syk and TLR/MyD88 pathways (which is extremely potent - see Eur J Immunol 38, 500–506 (2008)). The reason for suspecting synergy is that If you look at Fig. 1, contrary to the authors' assertion, there is a massive reduction of response when using MyD88-deficient DCs, greater than the deficiency in Fig. 5 when using Syk-deficient DCs. The reason for stating it is a co-receptor is that, of course, it is not there in

TLR5-deficient cells (or, at least, when alone cannot signal in response to flagellin). So, in my view, the model in Fig. 7 is by no means proven and the whole message of the paper may be incorrect.

We largely agree with this assessment of our data and did not mean to be dogmatic in data interpretation during the previous round of revision. We actually agree with Rev1 and the Executive Editor that there are multiple ways that Syk/CARD9 can potentially intersect with TLR5-flagellin uptake to explain our data. In the previous manuscript we presented a simple model where Syk and CARD9 are directly downstream of TLR5. However, we agree that this interpretation may be incorrect. As noted by the Executive Editor, it is equally likely that a distinct "Syk/CARD9 coupled co-receptor" exists and that interaction with this receptor could explain the phenotype that we observe. We also agree that data in Figure 1 suggest at least some role for Myd88, despite the absolute requirement for Syk and CARD9 suggested by some of our experiments. The Executive Editor noted that, "the data are interesting and, if written appropriately, the paper would be a useful contribution". We thank the Editor for this additional opportunity and have now rewritten the manuscript in an attempt to equally address these competing models. In an effort to present a balanced interpretation of the data, we have revised Figure 7 to incorporate each of the alternative models put forward by Reviewer 1 and the Executive Editor.

Other issues brought up during review are also important: Fig. 2 is absolutely not convincing and the quantitation of pSyk by FACS makes no sense (% positive cells? the staining, if real, looks unimodal).

As noted in the previous response to review, we agree that pSyk staining by western blot or by flow cytometry are not completely convincing when examined individually. However, we do feel that our data represents the best staining possible with this antibody and is indeed similar to other published reports. In the original paper, we had presented both sets of data (western blots and FACs) and reviewers indicated that the flow data were stronger and recommended removing only the western blot data. At this point, it is not clear how we should proceed. If the Editor would rather that we remove all data examining pSyk, we would certainly be willing to do so. However, any reader would likely wonder why pSyk was not examined in our study. Our preference would be to include both data sets since, although they are not individually conclusive, they suggest a trend that is further supported by our Syk-knockout and inhibitor data.

Reviewer: 1

Comments to the Author
The authors have not addressed my concerns. Their comment that the reagents required to assess flagellin uptake and degradation are lacking are not valid.
The authors could:
1) obtain radiolabeled protein from bacterial cultures;

European Journal of Immunology

2) iodinate the protein;
or 3) tag the protein with a fluorescent label, e.g., an AlexFluor dye.
Without the requested data, the authors' conclusions are simply not well-founded.

We agree with Reviewer 1 that additional interpretations of our data are possible and, as discussed above, we have now tried to incorporate these competing interpretations into our revised manuscript. However, we strongly disagree with the Reviewer 1's assertion that simple approaches are available to examine flagellin uptake and degradation. Indeed, each of the suggestions put forward by the Reviewer above have been tried and found wanting. The structure of flagellin is such that it folds back upon itself and thus both the amino and carboxy-termini directly contact TLR5 and are required to ligate this receptor. Thus, altering the 3-dimentional configuration of this protein through modifications often disrupts receptor binding and reduces flagellin activity. We have tried adding various "tags" to the ends and central portion of the protein and found that this reduces TLR5-dependent functional effects in vitro and in vivo. Indeed, we have tried this approach with GFP, RFP, and have also attempted to conjugate flagellin to fluorescent beads. Neither approach is of ANY value for examining TLR5-mediated uptake since TLR5-dependent functional activity was abrogated.
While we agree that the issue of flagellin uptake and degradation following TLR5 ligation is important and would help discriminate between various models of how TLR5 intersects with Syk and CARD9, we do not think this is essential to the main point of our manuscript, The central issue raised by our manuscript is that Syk and CARD9 are essential for DCs to present antigen to flagellin-specific T cells in a TLR5-dependent manner. This is a completely unexpected finding that has not been reported in the literature before. This finding is not undermined in any way by the technically challenging nature of interrogating flagellin uptake by DCs.

Reviewer: 2

The data presented in the revised manuscript support the conclusions claimed by the authors and have been strengthened by new data and statistics. The contribution and significance of the findings are objectively discussed. Their novelties justify a publication in EJI as they will lead to new ways to modulate antigen presentation of a widely represented bacterial component - i.e. flagellin - towards different applications in the context of vaccine design or to dampen chronic inflammations.

We thank the Reviewer for their constructive comments concerning the revised manuscript.

Minor points:
1/ There is a problem in figure 5 G that is an overlay of two of the same bar chart.

European Journal of Immunology

This has now been corrected in the revised figure.

2/ The same p value (p<0.05) is not associated consistently with the same symbols, sometimes *, ** or ***. These symbols could be removed as the p value is always specified on the figures.

We agree with the reviewer and have now removed these symbols.

Third Editorial Decision – 28 October 2014

Dear Dr. McSorley,

It is a pleasure to provisionally accept your manuscript entitled "Rapid CD4 T cell responses to bacterial flagellin require dendritic cell expression of Syk and CARD9" for publication in the European Journal of Immunology. For final acceptance, please follow the instructions below and return the requested items as soon as possible as we cannot process your manuscript further until all items listed below are dealt with.

Please note that EJI articles are now published online a few days after final acceptance (see Accepted Articles: http://onlinelibrary.wiley.com/journal/10.1002/(ISSN)1521-4141/accepted). The files used for the Accepted Articles are the final files and information supplied by you in Manuscript Central. You should therefore check that all the information (including author names) is correct as changes will NOT be permitted until the proofs stage.

We look forward to hearing from you and thank you for submitting your manuscript to the European Journal of Immunology.

Yours sincerely,
Karen Chu

on behalf of Prof. Caetano Reis e Sousa

Dr. Karen Chu
Editorial Office
European Journal of Immunology
e-mail: ejied@wiley.com
www.eji-journal.eu
